# Improving protein succinylation sites prediction using embeddings from protein language model

**DOI:** 10.1038/s41598-022-21366-2

**Published:** 2022-10-08

**Authors:** Suresh Pokharel, Pawel Pratyush, Michael Heinzinger, Robert H. Newman, Dukka B. KC

**Affiliations:** 1 Department of Computer Science, Michigan Technological University, Houghton, MI USA; 2 Department of Informatics, Bioinformatics and Computational Biology - i12, TUM (Technical University of Munich), Boltzmannstr. 3, 85748 Garching/Munich, Germany; 3 Center of Doctoral Studies in Informatics and Its Applications (CeDoSIA), TUM Graduate School, Boltzmannstr. 11, 85748 Garching, Germany; 4 Department of Biology, College of Science and Technology, North Carolina A&T State University, Greensboro, NC USA; 5 Department of Chemistry, University of North Carolina at Chapel Hill, Chapel Hill, NC USA

**Keywords:** Computational biology and bioinformatics, Machine learning, Protein function predictions

## Abstract

Protein succinylation is an important post-translational modification (PTM) responsible for many vital metabolic activities in cells, including cellular respiration, regulation, and repair. Here, we present a novel approach that combines features from supervised word embedding with embedding from a protein language model called ProtT5-XL-UniRef50 (hereafter termed, ProtT5) in a deep learning framework to predict protein succinylation sites. To our knowledge, this is one of the first attempts to employ embedding from a pre-trained protein language model to predict protein succinylation sites. The proposed model, dubbed LMSuccSite, achieves state-of-the-art results compared to existing methods, with performance scores of 0.36, 0.79, 0.79 for MCC, sensitivity, and specificity, respectively. LMSuccSite is likely to serve as a valuable resource for exploration of succinylation and its role in cellular physiology and disease.

## Introduction

Post-translational modifications (PTMs) are important regulators of proteins that modulate a myriad of physiological and pathological processes such as signal transduction, gene expression, metabolism, DNA repair, and cell cycle progression among many others^1^. Among more than 400 known PTMs, succinylation has emerged as an important PTM that has been implicated in numerous diseases such as hepatic, cardiac, pulmonary and neurological disorders^2^. Therefore, it is important to identify succinylation sites and how they affect protein function. Indeed, a better understanding of succinylation could facilitate the development of novel therapeutic compounds to treat diseases. However, comprehensive identification of succinylation sites, as well as understanding of its functional impact, remains elusive.

Succinylation, which is among the more recently discovered PTMs, is comparatively unique^3^. Like methylation, acetylation or ubiquitination, succinylation also occurs on lysine residues. However, compared to methylation (14 Da) or acetylation (40 Da), succinylation (100 Da) causes a larger mass change and converts a positively charged side chain to a negatively charged one, causing a two-unit charge shift in the charge of the modified residues^4^. During succinylation, a metabolically derived succinyl CoA modifies protein lysine residues. It has been shown that succinylation alters the catalytic rates of enzymes and the pathways in which they are involved, especially mitochondrial metabolic pathways^4^. This provides an elegant mechanism to coordinate metabolism and signaling. Additionally, succinylation is known to provide a link between metabolism and protein function in the nervous system and in neurological diseases, rendering deeper understanding of its mechanism highly interesting^4^. Likewise, succinylation has also been shown to be substantially upregulated in the early phases of infection by severe acute respiratory syndrome coronavirus 2 (SARS-CoV-2), suggesting that succinylation inhibitors may be able to reduce viral replication to treat COVID-19^5^. Interestingly, succinylation is observed in diverse organisms, including bacteria, yeast, and mouse and these sites are also frequent targets of acetylation (another important PTM)^6^. Given its widespread occurrence, and its implication in many diseases, characterization of succinylation is important for drug discovery.

Due to its widespread occurrence and its disease relevancy, various experimental techniques have been utilized to detect protein succinylation. Mass spectrometry (MS) has been one of the most popular experimental techniques for identifying PTMs, including succinylation^3^^,^^7–9^. For instance, Weinert et al.^6^ developed an antibody-based affinity enrichment of succinylated peptides followed by strong cation exchange (SCX) chromatography and liquid chromatography tandem MS (LC–MS/MS) to characterize succinylation sites.

Similarly, to bridge the gap between relatively sparse experimental data and the wealth of protein sequences in current databases, a plethora of computational methods have been developed to predict protein succinylation sites over the last decade. For instance, various machine learning-based methods like iSuc-PseAAC^10^, iSuc-PseOp^11^, pSuc-Lys^12^, MDCAN-Lys^13^, HybridSucc^14^ have been proposed for succinylation site prediction. Likewise, a random forest (RF)-based approach called SuccinSite^15^, a position specific scoring matrix (PSSM)-based predictor called PSSM-Suc^16^, a secondary structure and PSSM-based approach called SSEvol-Suc^17^, and a logistics-based approach called GPSuc^18^ have been developed.

Recently, deep learning-based approaches have also been developed for prediction of protein succinylation sites^19^. CNN-SuccSite^20^ uses four feature encoding techniques as input to a convolutional neural network (CNN)-based architecture to predict succinylation sites. We also developed a deep learning-based approach that does not require hand-crafted feature extraction but instead uses techniques from natural language processing (NLP), such as supervised word embedding, to extract vector representations (embeddings) directly from protein sequences. Together with one-hot encoding, those embeddings are used as input to a CNN-architecture in DeepSuccinylSite^21^. Similarly, MDCAN-Lys^13^ uses a multilane dense convolutional attention network to predict succinylation sites.

Recently, language models (LMs) have emerged as a powerful paradigm to learn embeddings directly from large, unlabeled natural language datasets. This is achieved, for example, by reconstructing corrupted tokens within a sequence of tokens from the remaining, non-corrupted tokens within the same sentence. In contrast to uncontextualized word embeddings which will always return the same embedding for a word irrespective of the surrounding words, embeddings from LMs are contextualized in that they render the embedding dependent on the surrounding words. These advances are now being explored in proteins as well as through protein language models (pLMs)^22^. These deep language models are an exciting breakthrough in protein sequence modeling as they were shown to capture complex dependencies between protein residues^23^ based solely on training using large, but unlabeled, protein sequence databases^24,25^ (rather than sequences belonging to a specific protein family or task). Since large protein sequence databases represent vast data goldmines, recently various pLMs have been developed for distilling information from them^26^. This information can be transferred to other tasks, for example, by extracting the hidden states of the last layer of the pLM and using it as input to predict some protein properties. These pLMs^27–31^ were shown to learn features that can be used to better capture sequence relationships.

Similar to LMs, pLMs are usually trained by masking some parts (usually single amino acids) of the input protein sequence and reconstructing it from non-corrupted sequence context. Instead of using the final classification output during inference, LMs and pLMs use the output of the last hidden layers of the network as a means of representing a protein sequence as numerical vectors called embeddings. This allows information gathered from large but unlabeled protein sequence databases to be transferred to much smaller but labeled sequence data sets (via transfer learning). In order to distinguish the different types of embeddings used in this work, we dub one strategy “supervised word embedding” and the other “ProtT5”^32^. These embeddings have been used in various structural bioinformatics applications, including the prediction of secondary structure, subcellular localization, binding residues^33^ variant effects, or the identification of remote homologs^22,29,31,34^.

In this work, we utilize the embeddings derived from pLM ProtT5 (based on the NLP sequence-to-sequence model T5^35^ but trained on Big Fantastic Database (BFD) and fine-tuned on Uniref50^36^ instead of natural language) in conjunction with supervised word embedding to improve the prediction of succinylation sites in proteins. The proposed deep-learning approach, called LMSuccSite, combines supervised word embeddings with embeddings from ProtT5 using a simple neural network architecture. This strategy achieves or exceeds state-of-the-art performance in the benchmark dataset. To the best of our knowledge, this is the first work to use pLM-based embeddings for the prediction of succinylation sites specifically, and PTMs, in general.

## Dataset and methods

### Dataset

We used the dataset used during the development of DeepSuccinylSite^21^ to train and test our approach. This dataset consists of experimentally verified succinylation sites provided by Hasan et. al.^15^ that was originally obtained from the UniProtKB/Swiss-Prot^37^ database and the NCBI protein sequence database. First, these sequences were obtained and subjected to redundancy removal using a CD-hit^38^ algorithm with a cut-off similarity of 0.3 to any other proteins in the dataset. This resulted in 5009 succinylated sites from 2322 protein sequences. Subsequently, the sequences were randomly separated into a training set consisting of 2192 protein sequences and a testing set consisting of 124 proteins. The training and test set sequences contain 4755 and 254 succinylation sites, respectively. It should be noted that the input to the ProtT5 is the full protein sequence. However, input to the supervised word embedding is a window sequence created around the central residues (positive or negative sites) by flanking it with an equal number of residues to the left and the right.

Since five of the positive succinylation sites were around the N- or C-termini of the protein, we were unable to extract a window of size 33 (corresponding to the optimal window size obtained during the development of DeepSuccinylSite^21^) around those sites. For this reason, we excluded these five sites, resulting in 4750 succinylation sites. After fixing positive training and testing sequences, negative sets were then extracted from the same protein sequences. Specifically, all other lysine (K) residues from the same protein sequences that were not annotated as succinylated (i.e., positive sites) were considered negative succinylation sites. This resulted in 50,565 negative succinylation sites in the training set and 2977 negative succinylation sites in the test set. To deal with the imbalanced training set, we performed random under sampling on the negative training set to obtain the same number of negative sites (4750) as the number of positive sites in the training set. The independent test set was kept as it is. The final number of sites in this dataset is shown in Table 1. The dataset is provided in the GitHub repository at https://github.com/KCLabMTU/LMSuccSite. It is worth noting that some of the existing approaches use an imbalanced training set and use threshold-moving to handle the imbalanced dataset^18,39^.

**Table 1:** Dataset description of the training and independent test dataset.

Dataset type	Positive (succinylated)	Negative (non-succinylated)
Training data	4750	50,565
Training data (after balancing)	4750	4750
Benchmark independent test data	254	2977

### Feature encoding

Since ML/DL models are only capable of understanding inputs in numerical space, it is imperative to convert the protein sequences into vectorized representations (i.e., feature vectors). In fact, the quality of these features is directly proportional to the robustness of the predictive models. To address this, we leveraged two encoding approaches to identify the best representation of succinylation sites.

Similar to DeepSuccinylSite^21^, the first approach uses the representation based on the local interaction of amino acids in the vicinity of K residues, which is achieved by a supervised word embedding (obtained via Keras’s embedding layer)^21^. In this strategy, the features take into consideration the influence of upstream and downstream flanking residues within the window sequence (or peptide) centered around the site of interest. The other encoding approach employs a pLM-based transformer model called ProtT5^32^ that extracts a contextualized representation (aka embeddings) of the site of interest (i.e., K residues) from full protein sequences. We only extracted embeddings from the encoder-side of ProtT5 in half-precision as it was shown in previous work that this outperforms embeddings of ProtT5’s decoder-side. Please note that both methods directly take protein sequences as an input (supervised word embedding takes a window sequence, and ProtT5 takes the full protein sequence), eliminating the requirement for handcrafted or manual extraction of features. Each feature encoding is described below in detail.

#### Supervised word embedding

The first type of feature we used in this work attempts to capture the local information that is extracted using supervised word embedding obtained using Keras’s embedding layer. The embedding encoding used in this work is similar to the embedding encoding in DeepSuccinylSite^21^. Essentially, a window sequence centered around the site of interest (i.e., a K residue) with an equal number of residues upstream and downstream was taken as an input. Based on a comparison of various window sizes (corresponding to odd numbers ranging from 11 to 41) performed on a base architecture using tenfold cross-validation, a window size of 33 was chosen for subsequent development. The results of experiments performed in order to determine the optimal window size is given in Section C of the Supplementary Materials (Supplementary Figure C1 and Supplementary Table C1). This window size is similar to the optimal window size identified during the development of our previous predictor, DeepSuccinylSite^21^.

This supervised word embedding, which is obtained via Keras’s embedding layer, is able to capture the local information between amino acids within a fixed-sized window. Embedding layers in Keras work by treating peptides as documents and individual amino acids within that peptide as words. Initially, each amino acid in a peptide is represented by a unique integer and the embedding layer is initialized with random weights. Hence, a peptide of length ***n*** will be represented by a vector of length ***n***. The output is a dense vector of dimension ***n*** x ***m*** where ***m*** is the size of the predefined vocabulary. The output vector is dynamically updated in subsequent epochs during training in a backpropagation fashion. This supervised learning nature of embedding from a set of protein sequences allows the network to learn the semantic similarity of amino acids within the embedded vector space. Furthermore, each vectorized representation is an orthogonal representation in some other dimension, thus, preserving the semantic quality of individual amino acids. In practice, the shape of the output vector is a parameter to be set before training. We set the size of the vocabulary to 21 as in DeepSuccinylSite^21^ to address 20 canonical amino acids found in proteins, along with a virtual amino acid. Therefore, for a given peptide of length 33, supervised word embedding returns a dense vector of shape (33,21).

#### ProtT5 encoding

Transfer learning is a promising machine learning methodology that transfers the knowledge learned in data-rich problems to similar data-limited problems. Protein language models learn summary representation that can be used to distill the knowledge from a large dataset, which can be used to improve downstream function prediction through transfer learning. The second type of feature that we use in this work is based on a pLM that captures global contextual information. Unlike supervised word embedding, the inputs to these models are full-length protein sequences (i.e., no window), generating embeddings for all residues in a full-length protein sequence. The pLM we utilized is called ProtT5^32^. Leveraging teacher-forcing and span-generation, ProtT5, is pre-trained in a self-supervised manner on a massive dataset called UniRef50^36^ that consists of 45 × 10^6^ protein sequences. Specifically, ProtT5 was trained by teacher forcing, i.e., input and targets were fed to the model with inputs being corrupted protein sequences and targets being identical to inputs but shifted to the right (span generation with span size of 1 for ProtT5). It is based on Google’s t5-3b model^35^, which consists of a 24 layer encoder-decoder and has around 2.8 × 10^9^ learnable parameters.

In our work, we used the pretrained ProtT5^32^ model to encode the features. This model takes the overall protein sequence as an input and returns an embedding vector of dimension 1024 for each amino acid. Notably, the ProtT5 is a context-dependent encoding approach. Hence, for succinylation site prediction, using ProtT5, we utilized an embedding vector (length 1024) corresponding to the site of interest (in our case K residue).

### Machine learning model and deep learning models

Below, we define briefly the machine learning and deep learning models used in the study.

Random Forest (RF)^40^ classifier is an ensemble method consisting of a number of decision trees. The decision trees independently make the decision and the class predicted with the most votes is considered the final output of the Random Forest algorithm.

Support Vector Machines(SVM)^41^ is a supervised learning algorithm commonly used for both classification and regression problems. The SVM algorithm maps the data into some higher dimensional feature space such that hyperplanes can be constructed to separate the classes. It uses a kernel function to transform the input data into a higher dimension. The objective is to maximize the margin between the support vectors and the hyperplane.

Extreme Gradient Boosting (XGBoost)^42^ is an optimized version of Gradient Boosting that uses a decision tree-based ensemble method to solve classification and regression problems. It uses a gradient descent algorithm as the cost function with parallel processing, tree pruning, and regularization techniques.

CNN1D (1-Dimensional Convolutional Neural Network) is a variant of CNN that performs the convolution operation along one dimension. CNN1D is mostly used for text and 1D signal data. In this work, we implemented a CNN1D architecture along with max-pooling and dropout layers and trained on ProtT5-based features. CNN2D (2-Dimensional Convolutional Neural Network) uses a 2D convolution layer, where a 2D kernel convolves over a two-dimensional feature space similar to images. A long short-term memory (LSTM) is a variant of artificial neural network feedback connections. LSTM is commonly used for classifying or making predictions over time-series data. We used Tensorflow’s Keras API to implement these deep learning-based models (i.e., ANN, CNN1D, CNN2D, LSTM). The hyperparameters and other details are explained in section B (Supplementary Tables B1, B2, B3, B4, B5).

### Model architecture

Our proposed ensemble deep learning model, which we termed LMSuccSite, is designed to capture knowledge from the supervised word embedding (herein referred to as the embedding module) using the dataset for succinylation and unsupervised language models learned from a large dataset of proteins (obtained via ProtT5). Initially, a 2D-CNN-based architecture was used for supervised embedding features while an artificial neural network (ANN)-based module was used for ProtT5 features. Eventually, a meta-classifier based on an ANN using feature sets generated from the outputs of the individual classifiers was trained. Below, we describe the model architecture in detail.

#### Embedding module

**Figure 1:**
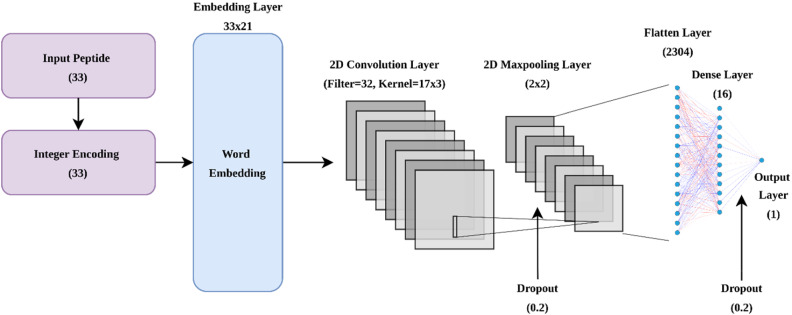
Architecture of supervised word embedding based model using a convolutional neural network.

The input to the embedding module is the output of the supervised word embedding, which is a dense vector of shape with dimensions of 33 and 21, where 33 represents the window size and 21 represents the size of the vocabulary. This module consists of a 2D convolutional layer, a 2D max-pooling layer, and a fully connected layer (consisting of a flatten layer, a dense layer, and an output layer). Two dropout layers were added to the network to avoid overfitting. Moreover, a rectified linear unit (ReLU) was used as an activation function in all the layers due to its representational sparsity and computational efficiency. The architecture of the embedding module is shown in Fig. 1. The optimization of parameters in the network was achieved using the Adam optimizer due to its combined benefits with respect to both adaptive gradient descent and root mean square propagation. Binary cross-entropy or log loss was used as the loss function for training. All the optimal hyperparameters used in the module are reported in Supplementary Table B3. This architecture is chosen based on tenfold cross-validation on the training set using different architectures with different combinations of hyperparameters using grid search.

#### ProtT5 module

**Figure 2:**
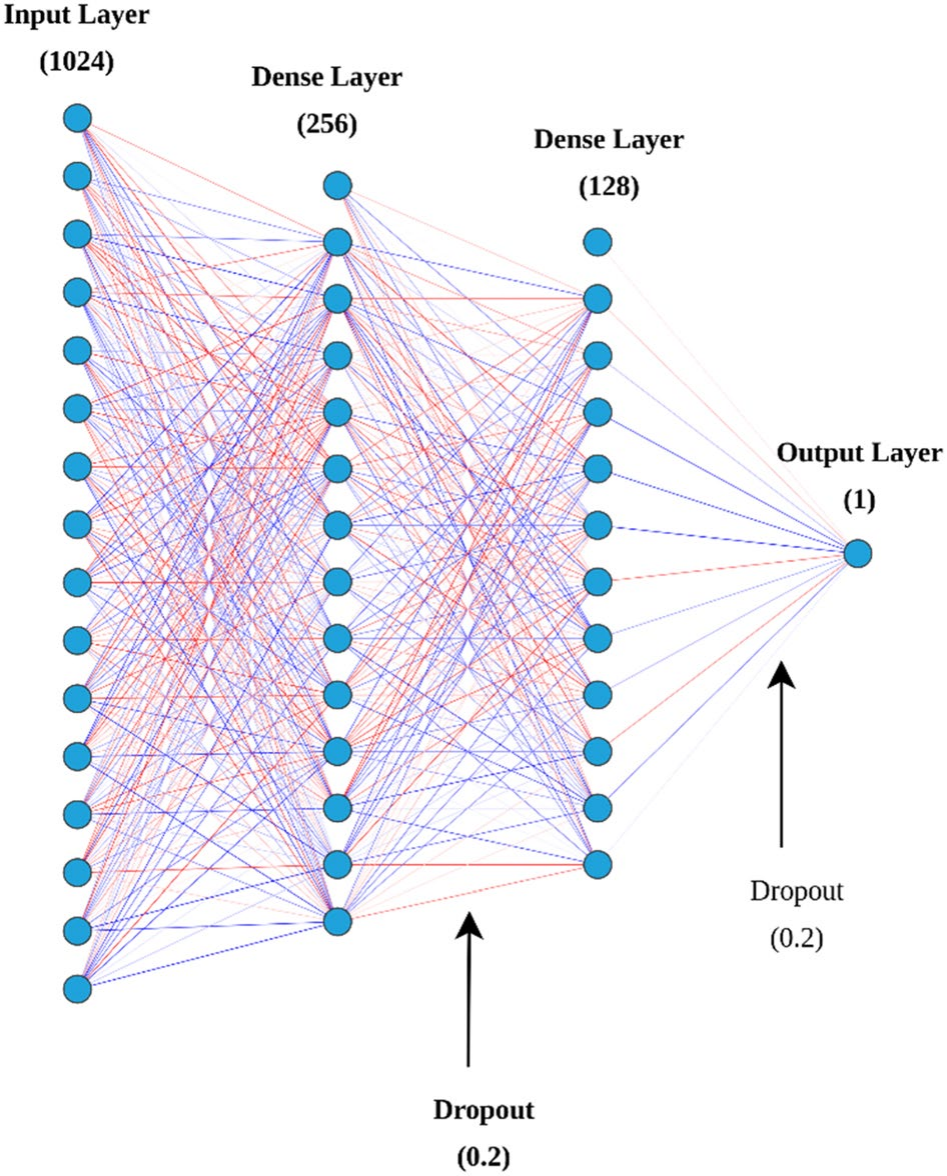
Architecture of Prot-T5 based model using a two-layer artificial neural network.

This module takes global contextual features extracted from the pLM ProtT5 for the residue of interest (i.e., K residue) as the input. The size dimension of the feature is 1024, as explained above. The ProtT5 module is based on an ANN architecture that consists of two hidden layers with sizes 256 and 128, each followed by a dropout layer. The architecture of ProtT5 based model is shown in Fig. 2. This architecture was chosen based on tenfold cross-validation on the training set using different machine learning and deep learning architectures with different combinations of hyperparameters using grid-search. The ReLU activation was used for both layers and the model was optimized using Adam based on binary cross-entropy loss. The parameters associated with this model are described in Supplementary Table S3 in supplementary materials. Similar to the work of Villegas-Morcello et al.^43^ and Weissenow et al.^23^, we also observed that these pLM-based features do not require a complex architecture to obtain competitive performance.

#### Meta-classifier

In order to combine the classifying capability of two different techniques, the embedding module and Prot-T5 module were combined using an ANN as a meta-classifier. Rather than using the final output of each individual module, we combined the learned features from the second-to-last layers from both modules (i.e., the last hidden layer in each module). During training, we paid special attention to the training process in order to avoid data leakage. To find the optimal architecture for the meta classifier, we performed tenfold cross-validation on the training set, ensuring that no data leakage occurred from the target information to the training set. In a classical stacked generalization^44^ methodology, the meta classifier might only learn from the predictions of the individual (base) models resulting in data leakage and over estimation of classification performance^45^.

Initially, all the layers of these two base modules (i.e., the embedding and ProtT5 modules) were frozen and the resulting features were obtained by concatenating the output (meta-feature) of the second-last layers from both modules. Importantly, the input size of the meta-classifier was 144 (16 from the embedding and 128 from the ProtT5 module). This meta-classifier is based on a simple feed forward neural network (NN) architecture. This architecture was chosen based on tenfold cross-validation experiments with different combinations of hyperparameters (e.g., number of hidden layers, number of neurons in each layer, regularization parameters) using grid-search. The hyperparameters used in the meta-classifier are provided in Supplementary Table S4. Furthermore, underfitting and overfitting in each module were carefully prevented by using early stopping.

**Figure 3:**
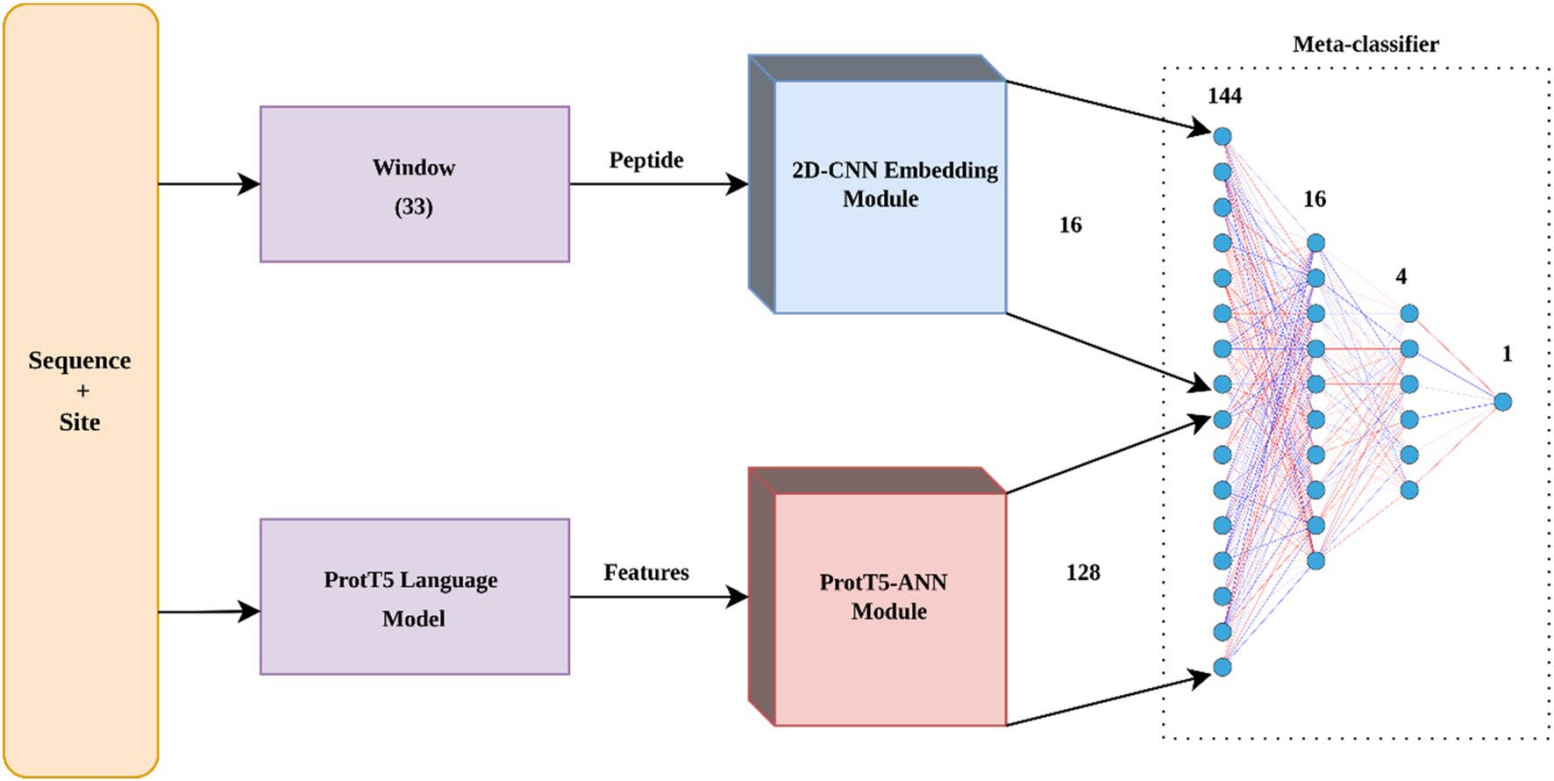
Model architecture of ensemble mechanism and meta classifier.

The final architecture of the meta classifier is simple, consisting of two hidden layers with ReLU activation and an output layer with softmax activation, as shown in Fig. 3. Similar to previous base modules, the meta classifier was also optimized using Adam based on binary cross-entropy loss.

### Performance evaluation

To evaluate the performance of the aforementioned models, we used the standard confusion matrix for binary classification that consists of four components: True Positives (TP), which represent the number of positive sites predicted as succinylated sites; True Negatives (TN), which correspond to the number of negative sites predicted as non-succinylated sites; False Positives (FP), which represent the number of negative sites predicted as succinylated sites; and False Negatives (FN), which describe the number of positive sites predicted as non-succinylated sites. Using these four components of the confusion matrix, evaluation metrics such as Accuracy (ACC), Matthew’s Correlation Coefficient (MCC), Sensitivity (Sn), Specificity (Sp), and geometric mean (g-mean) were calculated for each experiment. The detailed description of the performance metrics and equations are provided in Supplementary Table A1. We also used area under the receiver operating characteristic (AUROC) curve and Precision-Recall area under the curve (PrAUC) to further evaluate the discriminating ability of the models.

### T-distributed stochastic neighbor embedding

In order to further investigate the proposed models, we performed t-distributed stochastic neighbor embedding (t-SNE)^46^ visualization of learned features and the ablation study.

#### t-SNE visualization of learned features

The feature vector obtained from the final hidden layer can be projected into lower-dimensional latent cartesian space to visualize information available in high-dimensional space. To accomplish this, proteins or residues were colored according to a particular label. The clearer the distinction between the classes, the more readily available certain information is from the representation of sequences. In this regard, we utilized a t-SNE algorithm (with a perplexity of 30 and learning rate of 100) that can capture nonlinear signals within the data robustly, improving the visible boundary of separation. At first, the features generated from the embedding module and the ProtT5 module were visualized in 2-dimensional scatter plot using t-SNE to elucidate their respective boundary of separation between succinylated sites and non-succinylated sites. Then, the features learned from the ensemble model were projected onto a t-SNE to investigate if there was an improvement in the visible boundary of separation, which would indicate the usefulness of features obtained from the pLM.

#### Sensitivity analysis with respect to data size (Ablation study)

Sensitivity analysis is an intuitive technique to quantify the performance of a model under different inputs or varying environments. It can be performed by using various what-if analyses that tell how the changing input or other configuration affects the outcome. In this study, we analyzed the trend of model performance when the size of the training data gradually increases.

## Results

In this section, we describe the development and evaluation of LMSuccSite, a method that uses embedding from a pLM to predict protein succinylation sites in proteins using only the primary amino acid sequence as input. LMSuccSite combines two modules, one based on supervised word embedding and another based on a ProtT5 LM using a meta-classifier. First, we compare the performance of various DL/ML architectures for ProtT5 and supervised word embedding models using cross-validation techniques to find the best performing model. Subsequently, we compare the performance of various meta-classifiers for the stacked model using cross-validation to find the best meta-classifier. Finally, we compare the performance of our approach (LMSuccSite) with existing succinylation site prediction tools using an independent test set. The details are given in the following subsections.

### Training and evaluation of embedding module, ProtT5 module and LMSuccSite

As discussed in the method section, we performed tenfold cross-validation using the training set on the embedding and Prot-T5 modules for various ML/DL architectures (Table 2). Since the relative performance of various DL and ML based models for embedding has already been compared during the development of our previous model, DeepSuccinylSite^21^, we chose to utilize a similar architecture based on CNN2D. For the ProtT5 module, we implemented RF, support vector machines (SVM), XGBoost, CNN1D, and ANN architectures. As can be seen in Table 2, the CNN2D and ANN architectures exhibit the best performance in terms of the embedding and ProtT5 modules, respectively. Since extensive validation of various ML and DL models was already performed during the development of DeepSuccinylSite^21^, we only provided the results of CNN2D and LSTM models for supervised word embedding.

**Table 2:** 10-Fold cross-validation results on the training set of Embedding module, ProtT5 module with different ML and DL models.

Encoding approach	Architecture	ACC	MCC	Sn	Sp
Embedding	CNN2D	0.73 ± 0.02	**0.47 ± 0.05**	0.76 ± 0.01	0.70 ± 0.01
	LSTM	0.71 ± 0.01	**0.43** ± **0.02**	0.77 ± 0.04	0.66 ± 0.03
ProtT5	RF	0.62 ± 0.01	0.25 ± 0.01	0.59 ± 0.01	0.65 ± 0.01
	SVM	0.73 ± 0.01	0.46 ± 0.01	0.75 ± 0.02	**0.71 ± 0.01**
	XGBoost	0.70 ± 0.01	0.41 ± 0.01	0.76 ± 0.01	0.65 ± 0.01
	CNN1D	0.69 ± 0.01	0.38 ± 0.03	**0.78 ± 0.08**	0.59 ± 0.09
	ANN	**0.74 ± 0.01**	**0.47 ± 0.02**	0.76 ± 0.02	0.71 ± 0.02

The highest values in each category are bolded.

Based on these results, a CNN2D-based architecture performed the best in the category of supervised word embedding while ANN performed the best in the category of embedding from ProtT5. Hence, these two modules are combined using a meta-classifier. In order to determine the best architecture for the ensemble model, we performed tenfold cross validation using various architectures to combine these two best individual models.

We also used a class weight method for cost-sensitive learning for both Embedding and ProtT5 encoding schemes to train on an imbalanced dataset that includes all available positive and negative sites. Since the results of the experiments on an imbalanced dataset were not on par with the results using the balanced dataset, we used the balanced dataset in our subsequent experiments. The performance of different models trained on the imbalanced dataset using the class weighting method is presented in Table D1 (Supplementary Materials).

As discussed in the Methods section, the hyperparameters of the final stacked generalization model is obtained using grid search. The tenfold cross-validation results using various architectures for the best model (in terms of MCC) is shown in Table 3.

**Table 3:** Performance of best different ML/DL models as a meta classifier.

Encoding	Model	ACC	MCC	Sn	Sp
Embedding + ProtT5	SVM	0.76 ± 0.01	0.52 ± 0.02	0.80 ± 0.02	0.71 ± 0.02
	RF	0.75 ± 0.01	0.51 ± 0.02	0.79 ± 0.01	0.71 ± 0.02
	LR	0.74 ± 0.01	0.50 ± 0.03	0.78 ± 0.02	0.71 ± 0.02
	XGBoost	0.73 ± 0.02	0.46 ± 0.04	0.75 ± 0.03	0.71 ± 0.02
	ANN(LMSuccSite)	**0.77 ± 0.01**	**0.56 ± 0.02**	**0.80 ± 0.01**	**0.76 ± 0.02**

Highest values in each category are in [bold].

**Figure 4:**
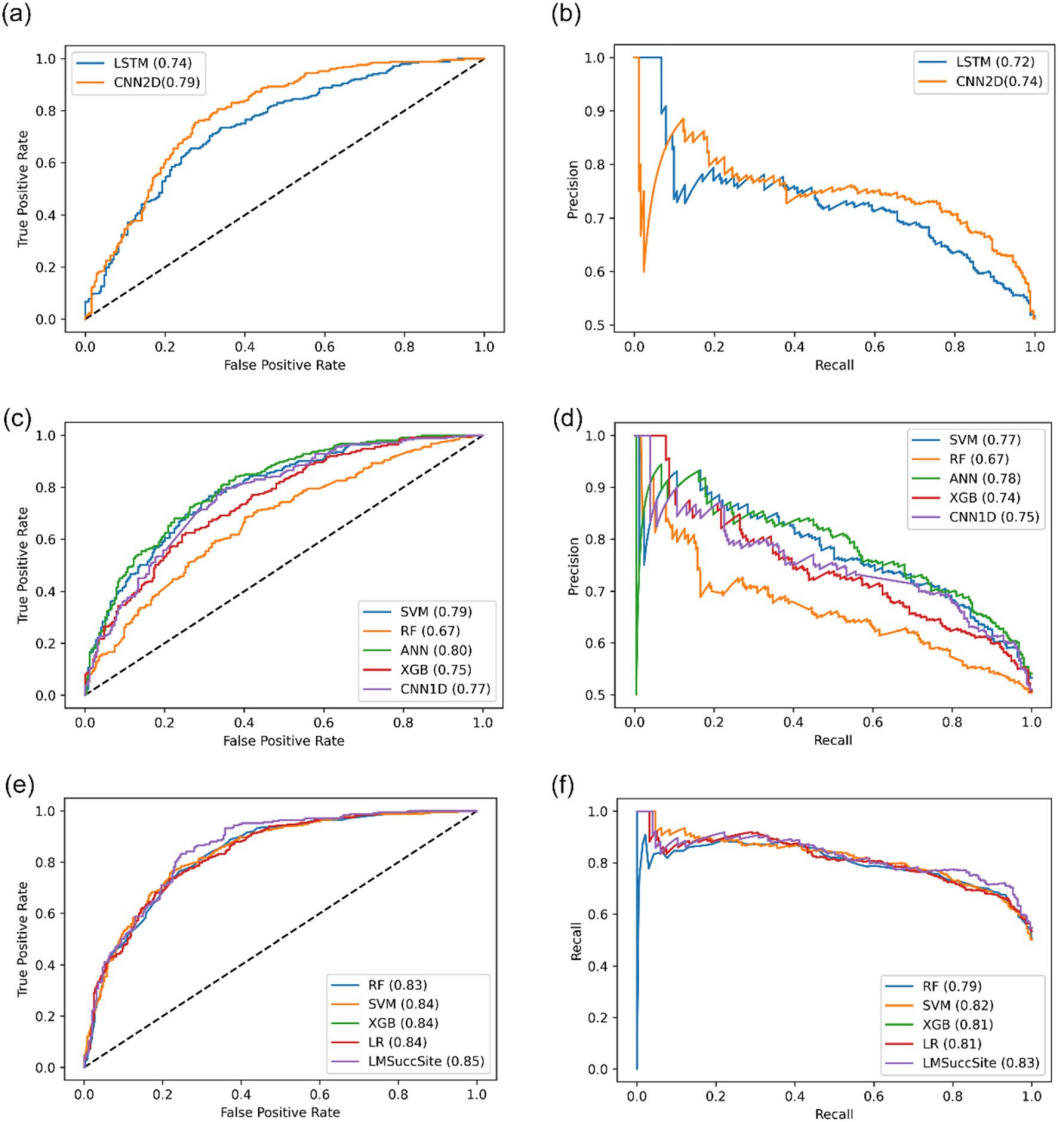
ROC (Receiver Operating Characteristic) curve and PR (Precision Recall) curves with AUC (Area Under Curve) for different models explained in Tables 3, 4. (**a**) ROC curves for supervised embedding-based models (**b**) PR curve for supervised embedding based models (**c**) ROC curves for Prot-T5 based models (**d**) PR curves for Prot-T5 based models (e) ROC curves of different meta classifier for combined models (**f**) PR Curves of different meta classifier for combined models.

We also compared the performance of these various models using the ROC curve for the tenfold cross-validation. The ROC and Pr-Auc curves of the embedding-based CNN2D model, different ProtT5-based models, and the combined model of ProtT5-based ANN and Embedding based 2DCNN are shown in Fig. 4. These data suggest that the AUC of the ANN-based combined model is better than those of the individual models. Hence, this model was chosen as the final model, which we termed LMSuccSite. Additionally, LMSuccSite was trained on the overall training data before being compared against other existing approaches.

### Comparison of LMSuccSite with other predictors

In order to compare the performance of LMSuccSite with the existing succinylation site predictors, we used the independent test set described in Table 1 and computed parameters such as accuracy, MCC, sensitivity, specificity and g-mean (Table 4). Importantly, the same training and test sets were used for all of the models tested. The results for other predictors were obtained from the respective articles.

**Table 4:** Comparison of our model with existing succinylation prediction tools using an independent test set.

Tool	ACC	MCC	Sn	Sp	g-mean
iSuc-PseAAC^10^	0.83	0.01	0.12	0.89	0.33
iSuc-PseOpt^11^	0.72	0.04	0.30	0.76	0.48
pSuc-Lys^12^	0.78	0.04	0.22	0.83	0.43
SuccineSite^15^	0.84	0.19	0.37	0.88	0.57
SuccineSite2.0^47^	0.85	0.26	0.40	**0.88**	0.63
GPSuc^18^	**0.85**	0.30	0.50	0.88	0.66
PSuccE^39^	0.85	0.20	0.38	0.89	0.58
DeepSuccinylSite^21^	0.70	0.27	**0.79**	0.69	0.74
LMSuccSite	0.79	**0.36**	**0.79**	0.79	**0.79**

The numbers are rounded to two significant digits after the decimal. The highest value in each category is shown in bold. *ACC* Accuracy, *MCC* Matthew’s Correlation Coefficient, *Sn* Sensitivity, *Sp* Specificity, *g-mean* geometric mean.

As observed in Table 4, LMSuccSite exhibited the highest MCC, Sn, and g-mean scores among the methods tested. Moreover, though it did not achieve the highest ACC and Sp scores, LMSuccSite still performed well in these areas, with ACC and Sp scores that were within 10 and 12% of the best performing methods, respectively. In terms of MCC (which is often used as a measure of overall method performance because it takes into account both Sn and Sp), LMSuccSite achieved a 22% increase in MCC compared to the next best succinylation site predictor, GPSUc^18^ (0.36 vs. 0.30).

As the performance of some existing tools have been evaluated by fixing specificity at different values (for example, the SuccinSite2.0 threshold was set at 0.9 specificity while iSuc-PseAAC set a cutoff threshold of 0.35), we also calculated Acc, Sn, and MCC of LMSuccSite at specificity values of 0.85, 0.90, and 0.95. The results are presented in Supplementary Table D3.

### Further analysis of LMSuccSite

**Figure 5:**
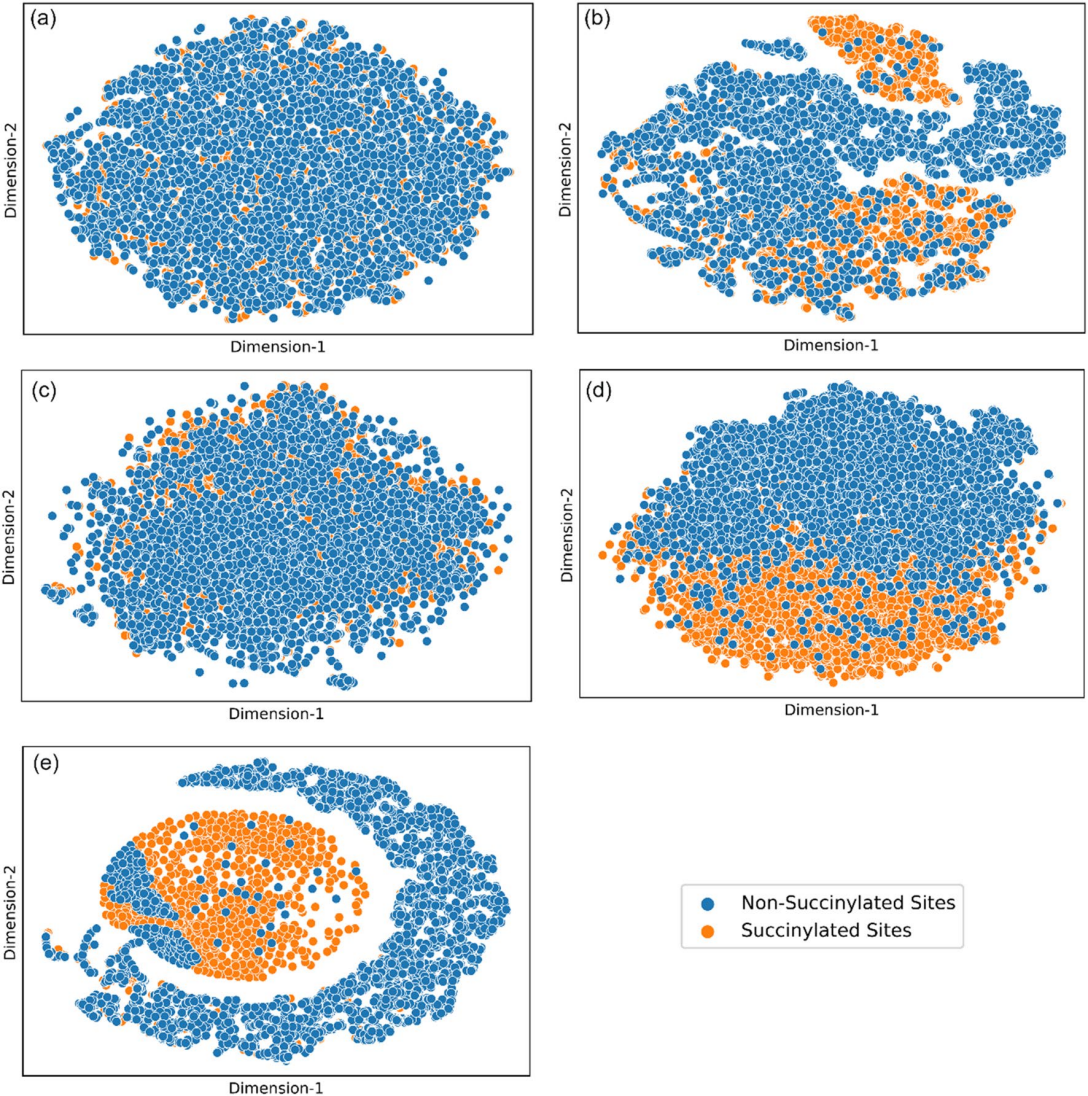
t-SNE to visualize the high-dimensional embedding learned by different features. (**a**) Before training CNN2D Embedding module (**b**) after training CNN2D with Embedding (**c**) before training the ANN model using prot-T5 features (**d**) after training the ANN model using prot-T5 features (**e**) after training the combined model. Blue dots represent non-succinylated sites and orange dots represent succinylated sites.

To gain insights into the basis for LMSuccSite’s improved performance, we then constructed t-SNE plots in R^2^ space for the original data and features learned from supervised word embedding, ProtT5 embedding, and the LMSuccSite model (Fig. 5). In contrast to the original data, the features learned from the embedded vector space show the formation of distinct clusters of succinylated (orange data points) and non-succinylated (blue data points) sites. This boundary of separation was even more pronounced when features were learned from the protT5 model, indicating that contextualized features are useful. Interestingly, the features learned from the ensemble model exhibited prominent distinctions between the clusters of succinylated sites and non-succinylated sites, as shown in Fig. 5.

#### Results of sensitivity analysis with respect to different training data size

**Figure 6:**
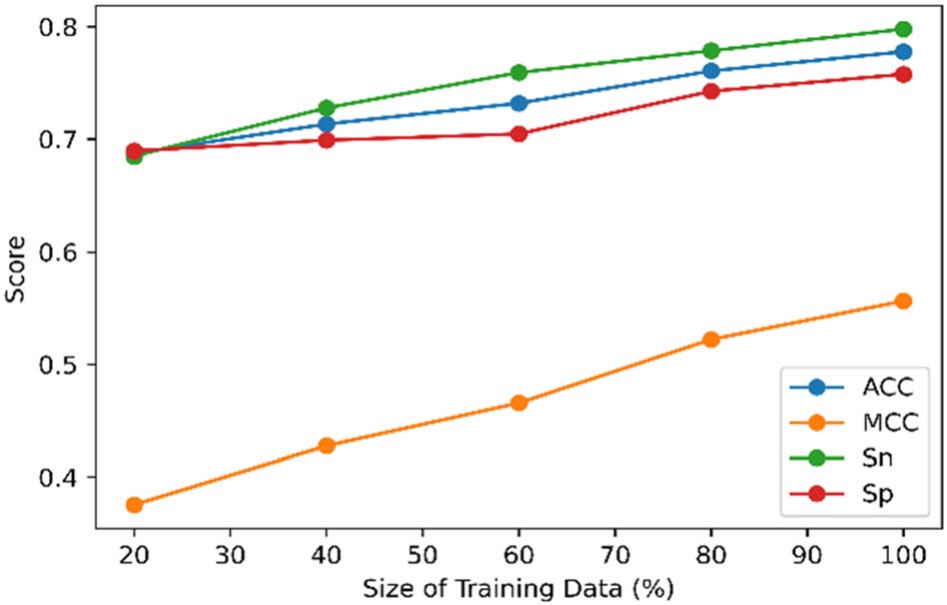
Sensitivity analysis of the final model on varying the size of available training data.

To explore the effects of training set size on model performance, we conducted sensitivity analysis (Fig. 6). To this end, we created four different training datasets by randomly selecting 20%, 40%, 60%, and 80% of the samples from our training set described in Table 1. Subsequently, tenfold cross-validation was performed in each of these random samples using our proposed model. These studies suggest that, as the size of training data increased, our model exhibited an increasing trend with respect to ACC, MCC, Sn, and Sp. It can be inferred that with the increase in the size of the training data, the results of our model will further improve.

## Conclusion and discussion

Succinylation, which is a recently discovered PTM, is garnering much interest due to the biological implications of introducing a relatively large (100 Da) chemical moiety that changes the charge of the modified residue. Given its widespread occurrence and its putative association with many diseases, including SARS-CoV-2 infection and the recent COVID-19 pandemic, the characterization of succinylation sites has important implications for drug discovery of many diseases as well. In conjunction with technological advances in experimental technologies to identify succinylation sites, various complementary computational approaches have been proposed for prediction of protein succinylation sites.

The available dataset for succinylation site is still relatively small. In the cases where the dataset is scarce, transfer learning becomes very useful. In particular, language models-based approaches that learn summary representations can be used to distill the knowledge from a large dataset and this knowledge can then be used to improve downstream prediction through transfer learning. In that regard, due to the availability of pLMs, it is now possible to use LMs that learn summary representation for feature extraction. In this regard, LMSuccSite uses embedding learned from the ProtT5 LM, which is trained on a T5 model on UniRef50^36,48^ protein dataset. By combining the embedding learned from a pLM with supervised word embedding using a meta-classifier on the existing succinylation dataset, we obtained overall improvement in the prediction of protein succinylation sites. This combination of supervised word embedding and embedding learned from pLM produced the best results, suggesting that contextual information obtained from pLMs is important for the prediction of succinylation sites.

Although pLMs have been used for other structural bioinformatics tasks, to the best of our knowledge this strategy of using embeddings from pLMs has not been explored for PTM site prediction, in general, and protein succinylation site prediction, in particular. Our data suggest that pLM-based representations are versatile features that can be used for various structural bioinformatics tasks. In addition, similar to the work of Villegas-Morcello et al.^43^ and Weissenow et al.^23^, we also observed that there is no requirement for complex architecture for these LM-based features to obtain competitive performance, as evidenced by the LMSuccSite architecture.

Interestingly, through t-SNE visualization, we found that the separation between succinylation and non-succinylation sites for the embedding learned from LMs shows clear boundaries. This boundary becomes more discernible when the features learned from the ensemble model show a prominent distinction between the clusters of succinylated sites and non-succinylated sites. Additionally, through sensitivity analysis, it can be inferred that LMSuccSite’s performance is likely to improve with the availability of more training data.

Recently, numerous LMs have been proposed for learning representation from a vast number of protein sequences. In that regard, comparative performance of various LMs for protein succinylation sites will be an important future work.

## Supplementary Information

Supplementary Information.

## Data Availability

The source code and trained models are publicly available in the GitHub repository at https://github.com/KCLabMTU/LMSuccSite. All the steps to extract the features and executing the programs are discussed in the same GitHub repository.

## Abbreviations

PTM: Post-translational modification
LMs: Language models
pLMs: Protein language models
DNA: Deoxyribonucleic acid
CoA: Coenzyme
MS: Mass spectrometry
SCX: Strong cation exchange (SCX)
CNN: Convolutional neural network
NLP: Natural language processing
BFD: Big fantastic database
T5: Text-to-text transfer transformer
ML: Machine learning
DL: Deep learning
ANN: Artificial neural network
NN: Neural network
2D: Two dimensional
ReLU: Rectified linear unit
TP: True positive
TN: True negative
FP: False positive
FN: False negative
ACC: Accuracy
MCC: Matthew’s correlation coefficient
Sp: Specificity
Sn: Sensitivity
AUROC: Area under receiver operating characteristics
PrAUC: Precision recall area under curve
T-SNE: T-distributed stochastic neighbor embedding
CNN2D: 2 Dimensional CNN
CNN1D: 1 Dimensional CNN
SVM: Support vector machine
RF: Random forest
XGBoost: Extreme gradient boosting
