# Supplementary material for: Improving protein succinylation sites prediction using embeddings from protein language model
Source: Sci Rep. 2022 Oct 8;12:16933. doi: 10.1038/s41598-022-21366-2 (PMC9547369; doi:10.1038/s41598-022-21366-2)
Supplement: Supplementary file 1 — Supplementary Information. [file 41598_2022_21366_MOESM1_ESM.docx]

# **Improving Protein Succinylation Sites Prediction Using Embeddings from Protein Language Model**

Suresh Pokharel^1^, Pawel Pratyush^1^, Michael Heinzinger^2,3^, Robert H Newman^4^, Dukka KC^1*^

**Supplementary Materials**

## Section A: Performance Metrics

Supplementary Table A1: Formula for the performance evaluation metrices

(TP = True Positive, TN = True Negative, FP = False Positive, FN = False Negative)

| **Metric** | **Description** | **Equation** |
| --- | --- | --- |
| Accuracy | Ratio between the number of correctly classified samples and the overall number of samples | $\frac{(TP+TN)}{(TP+TN+FP+FN)}$ |
| Sensitivity | Sensitivity is also called True Positive Rate (TPR). It is the probability of a positive test, conditioned on the result truly being positive. | $\frac{TP}{TP+FN}$ |
| Specificity | Specificity is also called True Negative Rate (TNR). It is the probability of a negative test, conditioned on the result truly being negative. | $\frac{TN}{TN+FP}$ |
| MCC | Matthew’s Correlation Coefficient (MCC) is a reliable performance metric for the imbalanced dataset. Better MCC scores are achieved if the model performs better in all four components of the confusion matrix (i.e., true positives, false negatives, true negatives, and false positives). | $\frac{TP*TN -FP*FN}{(TP+FP)(TP+FN)(TN+FP)(TN+FN)}$ |
| GMean | Geometric Mean (G-Mean) measures the balance between classification performances in positive and negative classes. | $\sqrt{Sensitivity*Specificity}$ |

## Section B: Hyperparameters for different ML/DL models

For deep learning-based models, we manually searched hyperparameters in a wide search space for different combination of hyperparameters, including the number of layers, number of neurons, dropouts, etc. The hyperparameters of those different deep learning-based models are provided in Tables B1, B2, B3, and B4.

Supplementary Table B1: Hyperparameters of ProtT5-based ANN model

| **Parameters** | **Settings** |
| --- | --- |
| Learning Rate | 0.001 |
| Intermediate Layer Activation Function | ReLU |
| Final Layer Activation Function | Sigmoid |
| Epochs | 60 |
| Batch Size | 256 |
| Dense | 256 |
| Dropout | 0.4 |
| Dense | 128 |
| Dropout | 0.4 |
| Callback | Earlystopping |

Supplementary Table B2: Hyperparameters of Embedding Based LSTM Model

| **Parameters** | **Settings** |
| --- | --- |
| Embedding Output Dimension | 21 |
| Learning Rate | 0.001 |
| Intermediate Layer Activation Function | ReLU |
| Final Layer Activation Function | Sigmoid |
| Batch Size | 256 |
| Epochs | 100 |
| LSTM | 64 |
| Dropout | 0.2 |
| Flatten | - |
| Dense 1 | 768 |
| Dropout | 0.5 |
| Dense 2 | 256 |
| Dropout | 0.5 |
| Callback | Earlystopping |

Supplementary Table B3: Hyperparameters of Embedding Based CNN2D Model

| **Parameters** | **Settings** |
| --- | --- |
| Embedding Output Dimension | 21 |
| Learning Rate | 0.001 |
| Intermediate Layer Activation Function | ReLU |
| Final Layer Activation Function | Sigmoid |
| Epochs | 100 |
| Conv2d_1 number of filters | 32 |
| Conv2d_1 filter size | 17 × 3 |
| Dropout | 0.2 |
| MaxPooling2d | 2 × 2 |
| Dense 1 | 16 |
| Dropout | 0.2 |
| Callback | Earlystopping |

Supplementary Table B4: Hyperparameters of ANN-based Combined Model

| **Parameters** | **Settings** |
| --- | --- |
| Learning Rate | 0.001 |
| Intermediate Layer Activation Function | ReLU |
| Final Layer Activation Function | Sigmoid |
| Batch Size | 256 |
| Epochs | 10 |
| Dense | 16 |
| Dense | 4 |
| Callback | Early stopping |

We used sklearn’s GridSearchCV method to tune hyperparameters of machine learning algorithms including SVM, RF, and XGBoost. This method performs k-Fold (we set k=10) cross validation for a given wide range of hyperparameters and finds the best parameters for that model.

The best parameters we found for ProtT5 based ML models are given in Table S10. All the hyperparameter names are based on sklearn library.

Supplementary Table B5: Hyperparameters of ProtT5 feature based machine learning models

| **Model name** | **Hyperparameters** |
| --- | --- |
| Random Forest | max_depth=20  max_features='sqrt'  min_samples_split=5  n_estimators=10 |
| Support Vector Machines | C=10  kernel='rbf' |
| XGBoost | seed = 321  max_depth = 10  learning_rate = 0.01  n_estimators = 100  colsample_bytree = 0.3 |

Supplementary Table B6: Hyperparameters for different metaclassifiers

| **Model name** | **Hyperparameters** |
| --- | --- |
| SVM | C = 3  gamma = 0.01  kernel = 'rbf' |
| RF | max_depth=15  max_features='sqrt'  min_samples_split=5  n_estimators=10 |
| LR | Sklearn’s default parameters |
| XGBoost | seed = 321  max_depth = 10  learning_rate = 0.01  n_estimators = 100  colsample_bytree = 0.3 |

## Section C: Finding optimal size of window

We tested embedding modules with different window sizes (odd numbers ranging from 11 to 41). As shown in the graph below, we found that window sizes 33, 35, and 37 exhibits almost similar performance in terms of 10-fold cross-validation MCC. Also considering the window size selection experiments of DeepSuccinylSite and MDCAN-Lys, we chose window size 33 for further experiments. The performance on different window sizes is illustrated in Supplementary Figure S1 and Supplementary Table C1.


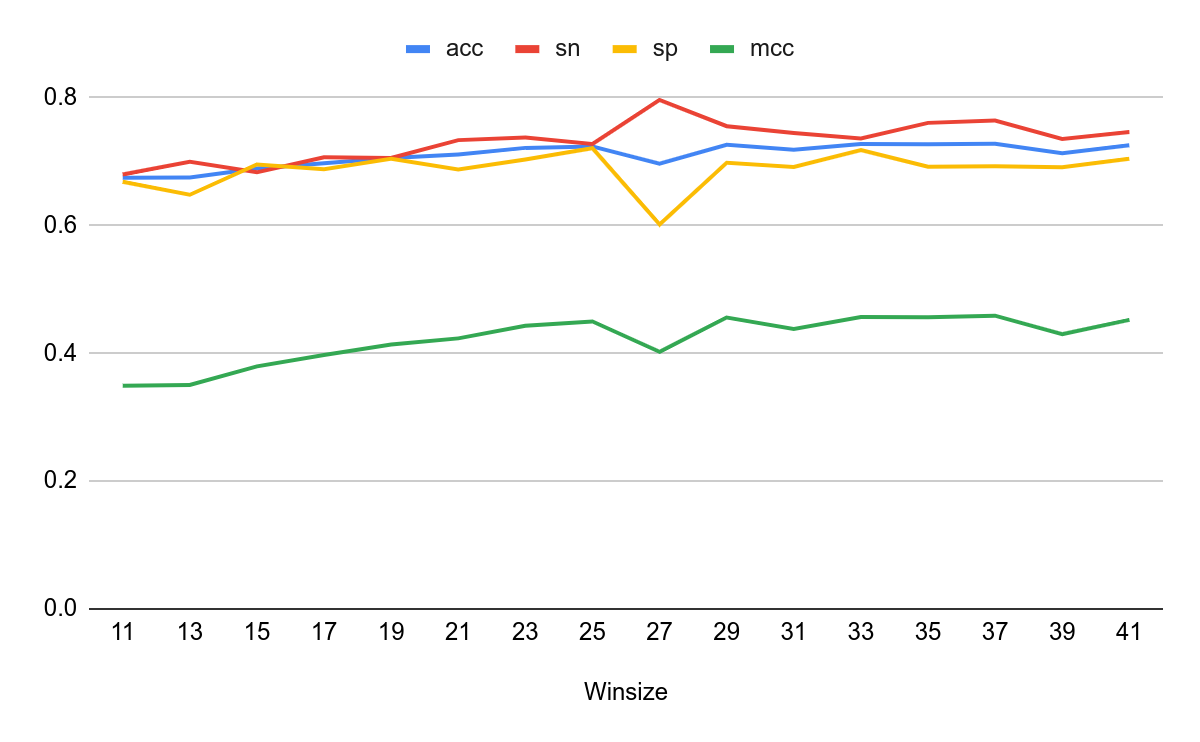


Supplementary Figure C1: Average of 10-Fold cross validation Accuracy(acc), Sensitivity(sn), Specificity(sp), and Matthew’s Correlation Coefficient(mcc) for different window sizes (Odd numbers from 11 to 41)

Supplementary Table C1: The average Accuracy (Acc), MCC, Sensitivity (Sn), and Specificity (Sp) of 10-fold Cross Validation results for different window sizes.

| **Winsize** | **Acc** | **MCC** | **Sn** | **Sp** |
| --- | --- | --- | --- | --- |
| 11 | 0.67 | 0.35 | 0.68 | 0.67 |
| 13 | 0.67 | 0.35 | 0.70 | 0.65 |
| 15 | 0.69 | 0.38 | 0.68 | 0.69 |
| 17 | 0.70 | 0.40 | 0.71 | 0.69 |
| 19 | 0.71 | 0.41 | 0.70 | 0.70 |
| 21 | 0.71 | 0.42 | 0.73 | 0.69 |
| 23 | 0.72 | 0.44 | 0.74 | 0.70 |
| 25 | 0.72 | 0.45 | 0.73 | 0.72 |
| 27 | 0.70 | 0.40 | 0.79 | 0.60 |
| 29 | 0.78 | 0.46 | 0.76 | 0.70 |
| 31 | 0.72 | 0.44 | 0.74 | 0.69 |
| 33 | 0.73 | 0.46 | 0.75 | 0.72 |
| 35 | 0.73 | 0.46 | 0.76 | 0.69 |
| 37 | 0.73 | 0.46 | 0.76 | 0.69 |
| 39 | 0.71 | 0.43 | 0.74 | 0.69 |
| 41 | 0.73 | 0.45 | 0.75 | 0.71 |

## Section D: Additional Results and Comparison

We trained Module-1 and Module-2 on the original imbalanced dataset mentioned in Table 1. The 10-fold cross-validation results of the individual models are shown in Table D1. Since the results on imbalanced training sets are generally of poorer quality than those generated using a balanced dataset and they take a great deal of training time compared to the balanced dataset, we used randomly undersampled data for subsequent experiments.

Supplementary Table D1: 10-fold cross validation results for Module-1 and Module-2 using imbalance data and balanced dataset

| **Dataset** | **Modules** | **ACC** | **MCC** | **SN** | **SP** |
| --- | --- | --- | --- | --- | --- |
| Imbalanced | Module-1 (Embedding) | 0.71 ± 0.02 | 0.29 ± 0.02 | 0.78 ± 0.03 | 0.71 ± 0.02 |
|  | Module-2 (ProtT5) | 0.74 ± 0.03 | 0.28 ± 0.01 | 0.71 ± 0.06 | 0.74 ± 0.04 |
| Balanced | Module-1 (Embedding) | 0.73 ± 0.02 | 0.47 ± 0.05 | 0.76 ± 0.01 | 0.70 ± 0.01 |
|  | Module-2 (ProtT5) | 0.74 ± 0.01 | 0.47 ± 0.02 | 0.76 ± 0.02 | 0.71 ± 0.02 |

Supplementary Table D2: Comparison of LMSuccSite with SuccineSite2.0 and GPSucc in terms of AUROC.

| **Tool’s Name** | **MCC** | **Sn** | **Sp** | **g-mean** | **AUROC** | **AUPRC** |
| --- | --- | --- | --- | --- | --- | --- |
| LMSuccSite | 0.36 | 0.79 | 0.79 | 0.79 | 0.86 | 0.31 |
| SuccineSite2.0 | 0.26 | 0.46 | 0.88 | 0.63 | 0.75 | - |
| GPSucc | 0.30 | 0.50 | 0.88 | 0.66 | 0.78 | - |

Supplementary Table D3**:** Comparison of LMSuccSite with other tools at different cutoff thresholds and fixed specificities on our independent test set

| **Tool’s Name** | **ACC** | **MCC** | **SN** | **SP** | **g-mean** |
| --- | --- | --- | --- | --- | --- |
| iSuc-PseAAC (cutoff threshold = 0.35) | 0.82 | 0.01 | 0.12 | 0.88 | 0.11 |
| SuccinSite2.0 (Sp = 0.90) | 0.85 | 0.26 | 0.40 | 0.88 | 0.57 |
| HybridSucc (Sp = 0.90) | 0.69 | 0.26 | 0.79 | 0.68 | 0.73 |
| LMSuccSite (Sp = 0.85, cutoff threshold = 0.70) | 0.84 | 0.35 | 0.66 | 0.85 | 0.75 |
| LMSuccSite (Sp = 0.90, cutoff threshold = 0.83) | 0.87 | 0.34 | 0.53 | 0.90 | 0.69 |
| LMSuccSite (Sp = 0.95, cutoff threshold = 94) | 0.90 | 0.27 | 0.31 | 0.95 | 0.53 |
| LMSuccSite(cutoff threshold = 0.5) | 0.79 | 0.36 | 0.79 | 0.79 | 0.79 |
